# The PET-Derived Tumor-to-Liver Standard Uptake Ratio (SUV_**TLR**_) Is Superior to Tumor SUVmax in Predicting Tumor Response and Survival After Chemoradiotherapy in Patients With Locally Advanced Esophageal Cancer

**DOI:** 10.3389/fonc.2020.01630

**Published:** 2020-09-03

**Authors:** Chunsheng Wang, Kewei Zhao, Shanliang Hu, Yong Huang, Li Ma, Minghuan Li, Yipeng Song

**Affiliations:** ^1^Department of Radiation Oncology, Qingdao University Medical College Affiliated Yantai Yuhuangding Hospital, Yantai, China; ^2^Department of Radiation Oncology, Shandong Cancer Hospital and Institute, Shandong University, Jinan, China; ^3^Department of Nuclear Medicine, Shandong Cancer Hospital and Institute, Shandong University, Jinan, China

**Keywords:** esophageal squamous cell carcinoma, SUVmax, tumor-to-liver SUVmax ratio, chemoradiotherapy, tumor response, survival

## Abstract

**Background:** The maximum standardized uptake values (SUVmax) derived from ^18^F-fluorodeoxy-glucose positron emission tomography/computed tomography (^18^F-FDG PET/CT) have some well-known shortcomings in predicting treatment response and prognosis in oncology. The standardized SUVmax with an appropriate reference background may overcome this problem in some instances. This study explored the prognostic value of the tumor-to-liver SUVmax ratio (SUV_TLR_) and the tumor-to-blood pool SUVmax ratio (SUV_TBR_) in predicting the objective response (OR) and overall survival (OS) in patients with locally advanced esophageal cancer after concurrent chemoradiotherapy (CCRT).

**Methods:** We retrospectively analyzed 128 newly diagnosed esophageal squamous cell carcinoma (ESCC) patients who were treated with CCRT. The SUVmax of primary tumor, SUV_TLR_, SUV_TBR_ and clinicopathologic features data were analyzed. Univariate and multivariate analyses were used to determine the predictors of tumor response. Survival analysis was performed using the Kaplan–Meier method and Cox proportional hazards model.

**Results:** Receiver operating characteristic (ROC) curve analysis demonstrated that SUV_TLR_ was superior to SUVmax and SUV_TBR_ in predicting treatment response. Univariate and multivariate analyses revealed that advanced tumor stage (hazard ratio [HR] = 9.67; 95% CI: 1.15-81.28; *P* = 0.037) and high SUV_TLR_ (HR = 21.92; 95% CI: 2.26-212.96; *P* = 0.008) were independent predictors of poor treatment response. Cox regression analysis showed that good clinical tumor response (*p* < 0.014, HR =0.501; 95% CI: 0.288–0.871) was a favorable independent predictive factor for OS, while an advanced tumor stage (p = 0.018, HR = 1.796; 95% CI: 1.107-2.915) and a high SUV_TLR_ (*p* < 0.002, HR = 2.660; 95% CI: 1.425–4.967) were prognostic factors for poor OS. The median OS of patients in the low SUV_TLR_ and high SUV_TLR_ groups was 13.47 vs. 19.30 months, respectively.

**Conclusions:** PET-derived SUV_TLR_ is superior to tumor SUVmax and SUV_TBR_ in predicting treatment response and overall survival in patients with ESCC undergoing CCRT. High SUV_TLR_ was an independent predictor of poor treatment response and shorter overall survival.

## Introduction

Esophageal cancer (EC) is the eighth most common malignancy and the sixth leading cause of cancer-related death worldwide (1). The extremely high mortality may be due to the fact that most patients with esophageal cancer have locally advanced disease at the time of diagnosis (2, 3). Concurrent chemoradiotherapy (CCRT) has been established as a first-line treatment for these patients with locally advanced esophagus cancer, according to the phase III intergroup trial of RTOG 85-01 (4). However, the treatment outcome of CCRT remains to be improved, despite CCRT improved local control and overall survival compared with radiotherapy alone. According to the literature, the overall response rate of CCRT in patients with esophageal cancer ranges from 53.3 to 94.0% (5–7), with an estimated 5-year overall survival (OS) rate varying from 20 to 31% (4, 7, 8). The assessment of tumor response and survival in advance plays an important role in the treatment of the disease (9).

^18^F-Fluorodeoxy-glucose positron emission tomography/computed tomography (^18^F-FDG PET/CT) is a functional imaging technique based on the theory that cancer cells generally exhibit increased glucose uptake and glycolysis. PET/CT is now widely used and is considered a useful imaging technique for cancer detection, staging, planning and monitoring treatment (10, 11). In addition, PET/CT is known to be effective for predicting treatment response and prognosis (12–14). The semiquantitative parameter derived from FDG-PET, maximum standardized uptake values (SUVmax), has been reported to be a strong predictor of survival in patients with esophageal cancer (13, 14). SUVmax has also been associated with CCRT response (15–17). Other volume-based PET parameters such as metabolic tumor volume (MTV) and total lesion glycolysis (TLG) are dependent on SUVmax. However, these PET parameters based on SUVmax methodology has some well-known shortcomings, such as weight, blood glucose level, time interval, and technical factors (17–20). The use of total weight is the most commonly used method of calculating the average radioactive concentration. However, heavier patients often have a higher body fat percentage, and white body fat is less metabolically active (i.e., takes up less FDG) than muscle tissue. Therefore, comparison of SUVs among patients with different body compositions is flawed (17). On the other hand, the fasting plasma glucose levels may vary significantly between examinations, especially in diabetic patients (21). In addition, different manufacturers and scanner models have different physical properties and different acquisition as well as reconstruction options. Each scanner has a calibration coefficient that converts the measured count to radioactivity. The calibration method and care of how this calibration is performed will affect the basic quantitative accuracy of PET scanner (17). Many recent studies have suggested that standardization of semiquantitative measurement may be superior to SUVmax in the prediction of treatment response and prognosis (22, 23). Standardization in its most common form is a ratio of FDG uptake in tumors to that in normal background tissue, such as the liver and mediastinal blood pool (24–28). This method can provide reliable and reproducible data across different PET scanners and improve the PET characterization of tumors more accurately (29–32).

However, data regarding the prognostic significance of the tumor-to-liver SUVmax ratio (SUV_TLR_) and tumor-to-blood pool SUVmax ratio (SUV_TBR_) in EC are limited. Hence, we conducted this retrospective study to evaluate the predictive value for treatment response and prognosis of SUV_TLR_ and SUV_TBR_ in patients with esophageal squamous cell carcinoma (ESCC) undergoing first-line chemoradiotherapy.

## Materials and Methods

### Patients

The ethics committee of Shandong Cancer Hospital and Institute approved the study. In addition, informed consent was exempted due to the retrospective nature of the study. Patients were included if they had an Eastern Cooperative Oncology Group (ECOG) performance status between 0 and 2 and were confirmed by histopathological analysis. They also needed to fulfill the following criteria: (1) adequate hematological, liver, and renal function; (2) completed PET/CT examination 1 week before treatment; and (3) locally advanced disease based on the 7th edition of the American Joint Committee on Cancer guidelines (AJCC 7th edition), with comorbidities making the tumor ineligible for surgery. The exclusion criteria were as follows: (1) distant metastasis or multiple primary esophageal lesions, (2) history of other malignancies, or (3) any liver disease that may affect liver metabolism and function, such as acute or chronic hepatitis, fatty liver, cirrhosis. We finally included 128 newly diagnosed ESCC patients who were treated with definitive CRT between January 2012 and December 2016 in our hospital.

### PET/CT Scanning and Image Analysis

PET/CT scanning was performed 1 week before treatment with an advanced PET/CT scanner (Discovery LS, GE Healthcare). Before undergoing PET/CT scans, all patients were asked to fast for at least 6 h and have a blood glucose level ≤ 11.1 mmol/L. Then each of they were injected into 3.5–4.5 MBq/kg of 18F-FDG. Sixty minutes later, a whole-body PET and CT scans were initiated from top of the skull to the proximal thigh for 5 min per field of view, each covering 14.5 centimeters, with an axial sampling at 4.25 millimeters per slice. The PET data sets were reconstructed with CT-derived attenuation correction using the ordered-subset expectation maximization algorithm. The attenuation-corrected PET images, CT images, and fused PET/CT images were displayed as coronal, sagittal, and axial slices on the Xeleris workstation (GE Healthcare). Two experienced nuclear medicine physicians visually and semiquantitatively analyzed the PET images by measuring the SUVmax of the primary tumor, the liver and the blood pool. Contour threshold method standard uptake values (SUVs) were based on the region of interest (ROI), which was a suspicious area showing an increased FDG uptake compared to the surrounding esophagus tissue. A threshold SUV of 2.5 was used to define ROI boundaries, which has been widely approved. The SUVmax of the liver and the blood pool was calculated using a round-shaped 10-mm ROI at the VIII hepatic segment and the aortic arch (without involving the vessel wall). Then the ratio of the SUVmax of the primary tumor to the SUVmax of the liver (SUV_TLR_) and the ratio of SUVmax of the primary tumor to the SUVmax of blood pool (SUV_TBR_) were calculated, respectively.

### Treatment Protocols

All patients received CCRT as their treatment option. The radiation treatments were performed using intensity-modulated radiation therapy (IMRT) with a total dose of 50–64 Gy administered once daily in 25–32 fractions (i.e., 1.8 or 2.0 Gy/ fractions, 5 days/week). Chemotherapy began on Day 1 concurrent with the initial radiation treatments, cycled every 28 days for 2–4 cycles for 2 cycles with radiation followed by 2 cycles without radiation. 5-Fluorouracil (700 mg/m^2^) was administered intravenously (iv) continuous infusion over 24 h daily on Days 1–4, and cisplatin (75 mg/m^2^) was administered by iv on Day 1.

### Assessment of Response and Follow-Up

Patients were asked to visit the clinic within 2–4 weeks after completion of all therapies. Barium meal and contrast-enhanced thoracic and abdomen computed tomography scans were used to evaluate treatment response based on evaluation criteria in solid tumors (RECIST) Version 1.1. Tumor response was defined as complete response (CR) or partial response (PR); non-response was defined as stable disease (SD) or progressive disease (PD). A primary tumor response that fulfilled the CR criteria and PR criteria was defined as objective response (OR) (OR = CR+PR), and the other was defined as Non-OR. These examinations were performed for all patients every 3 months for the first 2 years and every 6 months thereafter. The last follow-up date was December 30, 2018.

### Statistical Analysis

The selection of cut-off values of baseline PET/CT parameters was determined using receiver operating characteristics (ROC) curve analysis. Analysis of the AUCs of the ROC curves was done using Delong's test to compare the performance of PET/CT parameters for predictive response. Univariate and multivariate logistic regression analyses were used to determine the clinical tumor response predictors. OS was defined as the interval between the treatment and death or the last follow-up. The Kaplan-Meier method and the log-rank test were used to analyze the association of each marker with OS, and the associated 95% CIs were calculated. Cox's proportional hazards models were used to perform multivariate analysis defining the independent prognostic factors for OS, and hazard ratios were reported as relative risks with corresponding 95% confidence intervals. The analyses were performed with the SPSS 22.0 program (SPSS Inc., Chicago, IL, USA) and the MedCalc program (Version 18.11). A two-sided p-value < 0.05 was considered statistically significant.

## Results

### Patient Characteristics

A total of 128 newly diagnosed ESCC patients were retrospectively analyzed, including 103 (80.47%) males and 25 (19.53%) females, with a median age of 65 years (range: 39–83 years). Patients who had a history of smoking were slightly more represented (56.4%) than those who had never smoked, as well as those with a history of alcohol consumption. The lesions were mainly located in the middle thoracic segment of the esophagus (59, 46.10%), followed by the lower thoracic segment (35, 27.34%), and the upper thoracic segment (27, 21.09%), and lesions in the cervical segment were the least common (7, 5.47%). Additionally, most of the patients had stage III disease (96, 75.0%), whereas 32 (25.0%) had stage II disease. The number of patients in the OR and non-OR groups was 105 and 23, respectively, with an overall OR rate of 82.03%. The patients' clinical characteristics are summarized in Table 1.

**Table 1:** Patient baseline characteristics.

**Characteristics**	**Values**	**Percentage (%)**
**Age (years)**		
Median	65	
Range	39-83	
**Sex**		
Male	103	80.47
Female	25	19.53
**Smoking history**		
Yes	74	57.81
No	54	42.19
**Drinking history**		
Yes	68	53.13
No	60	46.87
**Tumor location**		
Cervical	7	5.47
Upper thoracic	27	21.09
Mid-thoracic	59	46.10
Lower thoracic	35	27.34
**T stage**		
1-3	101	78.91
4	27	21.09
**N stage**		
0	25	80.47
1-3	103	19.53
**Tumor stage**		
II	32	25.00
III	96	75.00
**Tumor response**		
OR	105	82.03
Non-OR	23	17.97

### ROC Curve Analysis

**Figure 1:**
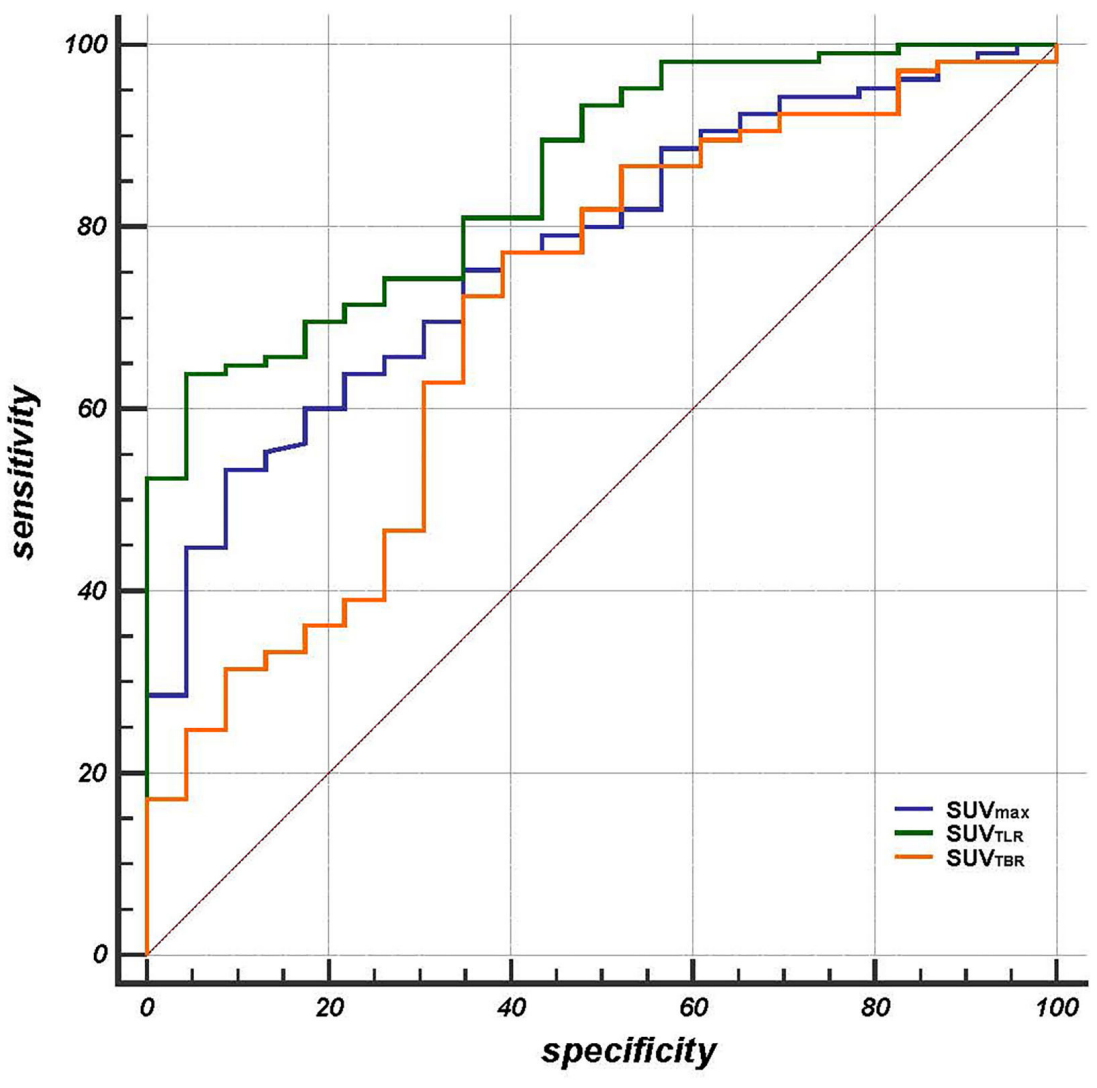
The area under the curve of SUV_TLR_ (AUC = 0.855 95% CI: 0.782-0.911) was significantly larger than that of SUVmax (AUC = 0.755, 95% CI: 0.693-0.844; ΔAUC = 0.0797, *p* = 0.018) and SUV_TBR_ (AUC = 0.705, 95% CI: 0.618-0.782; ΔAUC = 0.1500, *p* = 0.024).

The patients had a median SUVmax of primary tumor of 15.5 (range: 3.43–31.54), SUV_TLR_ of 4.04 (range: 0.73–9.09) and SUV_TBR_ of 3.79 (range: 0.30–14.59). All of the parameters that were calculated from PET/CT are summarized in Table 2. By ROC curve analysis, we found that SUV_TLR_ had the largest area under the curve of 0.855 (95% CI: 0.782–0.911), which was significantly larger than that of SUVmax (AUC = 0.755, 95% CI: 0.693–0.844; ΔAUC = 0.0797, *p* = 0.018) and SUV_TBR_ (AUC = 0.705, 95% CI: 0.618–0.782; ΔAUC = 0.1500, *p* = 0.024) (Figure 1). The optimal cut-off value for SUV_TLR_ was 4.21 (sensitivity 95.7%, specificity 63.8%), it was 14.68 for SUVmax (sensitivity 91.3%, specificity 53.3%), and 5.06 for SUV_TBR_ (sensitivity 60.9%, specificity 77.1%) (Table 3). Based on these cut-off values, patients were stratified into different PET/CT parameter groups. The baseline clinical characteristics based on PET/CT parameters are summarized in Supplementary Table 1.

**Table 2:** PET parameters.

**Variables**	**Mean**	**Range**
SUVmax of primary tumor	15.50	3.43–31.54
SUVmax of liver	3.93	2.39–5.79
SUVmax of blood pool	6.31	1.51–18.44
SUV_TLR_	4.04	0.73–9.09
SUV_TBR_	3.79	0.30–14.59

*SUV_TLR_, tumor-to-liver SUVmax ratio; SUV_TBR_, tumor-to-blood pool SUVmax ratio*.

**Table 3:** Analysis of ROC curves in predicting treatment response.

**Variable**	**AUC**	**SE**	**95%CI**	**ΔAUC**	***p***	**Cut-off**	**Sensitivity**	**Specificity**
SUVmax	0.775	0.0481	0.693-0.844	0.0797*	0.018^#^	14.68	91.3%	53.3%
SUV_TLR_	0.855	0.0378	0.782-0.911	0.150**	0.024^##^	4.21	95.7%	63.8%
SUV_TBR_	0.705	0.0622	0.618-0.782	0.0702***	0.281^###^	5.06	60.9%	77.1%

* and ^#^: The comparison of ΔAUC between SUVmax and SUV_TLR_.

** and ^##^: The comparison of ΔAUC between SUV_TLR_ and SUV_TBR_.

*** and ^###^: The comparison of ΔAUC between SUVmax and SUV_TBR_.

*SUV_TLR_, tumor-to-liver SUVmax ratio; SUV_TBR_, tumor-to-blood pool SUVmax ratio*.

### Univariate and Multivariate Analyses of Treatment Response

The univariate analysis revealed that tumor stage (*P* = 0.012), SUVmax (*P* < 0.001), SUV_TLR_ (*P* < 0.001) and SUV_TBR_ (*P* < 0.001) were prognostic factors for OR. Advanced tumor stage (stage III), high SUVmax (>14.68), high SUV_TLR_ (>4.21), and high SUV_TBR_ (>5.06) were significantly associated with poor treatment response. The objective remission rates of the tumor stage III group, high SUVmax group, high SUV_TLR_ group and high SUV_TBR_ group were 77.08, 68.57, 63.33, and 63.16%, respectively. However, these values were significantly higher in the corresponding groups (96.87, 96.55, 98.53, and 90.00%, respectively). However, none of the other parameters (i.e., age, sex, smoking history, drinking history, tumor location, T stage, and N stage) showed significant differences (Table 4). Subsequently, the significant variables from the univariate analysis (tumor stage, SUVmax, SUV_TLR_, and SUV_TBR_) were included in the multivariate logistic regression models. Multivariate analysis revealed that only advanced tumor stage (hazard ratio [HR] = 0.103; 95% CI: 0.012-0.87; *P* = 0.037) and high SUV_TLR_ (HR = 0.446; 95% CI: 0.074-2.692; *P* = 0.008) were independent predictors of poor treatment response (Figure 3).

**Table 4:** Univariate analysis for treatment response.

**Characteristics**	**Total *n* = 128**	**Tumor response**		***p***
		**OR (%)**	**Non-OR (%)**	
**Age**				
≤ 60	44	34 (77.27)	10 (22.73)	0.310
>60	84	71 (84.52)	13 (15.48)	
**Sex**				
Male	103	84 (81.55)	19 (18.45)	0.775
Female	25	21 (84.00)	4 (16.00)	
**Smoking history**				
Yes	74	62 (83.78)	12 (16.21)	0.545
No	54	43 (79.63)	11 (20.37)	
**Drinking history**				
Yes	68	56 (82.35)	12 (17.65)	0.920
No	60	49 (81.67)	11 (18.33)	
**Tumor location**				
Cervical	7	4 (57.14)	3 (42.86)	0.264
Upper thoracic	27	22 (81.48)	5 (18.52)	
Mid-thoracic	59	48 (81.36)	11 (18.64)	
Lower thoracic	35	31 (88.57)	4 (11.43)	
**T stage**				
1-3	101	86 (85.15)	15 (14.85)	0.076
4	27	19 (70.37)	8 (29.63)	
**N stage**				
0	25	23 (92.00)	2 (8.00)	0.148
1-3	103	82 (79.61)	21 (20.39)	
**Tumor stage**				
II	32	31 (96.87)	1 (3.13)	0.012
III	96	74 (77.08)	22 (22.92)	
**SUVmax**				
≤ 14.68	58	56 (96.55)	2 (3.45)	<0.001
>14.68	70	38 (68.57)	22 (31.43)	
**SUV**_**TLR**_				
≤ 4.21	68	67 (98.53)	1 (1.47)	<0.001
>4.21	60	38 (63.33)	22 (36.67)	
**SUV**_**TBR**_				
≤ 5.06	90	81 (90.00)	9 (10.00)	<0.001
>5.06	38	38 (63.16)	14 (36.84)	

*SUV_TLR_, tumor-to-liver SUVmax ratio; SUV_TBR_, tumor-to-blood pool SUVmax ratio*.

### Prognostic Values of the PET/CT Parameters for OS

**Figure 2:**
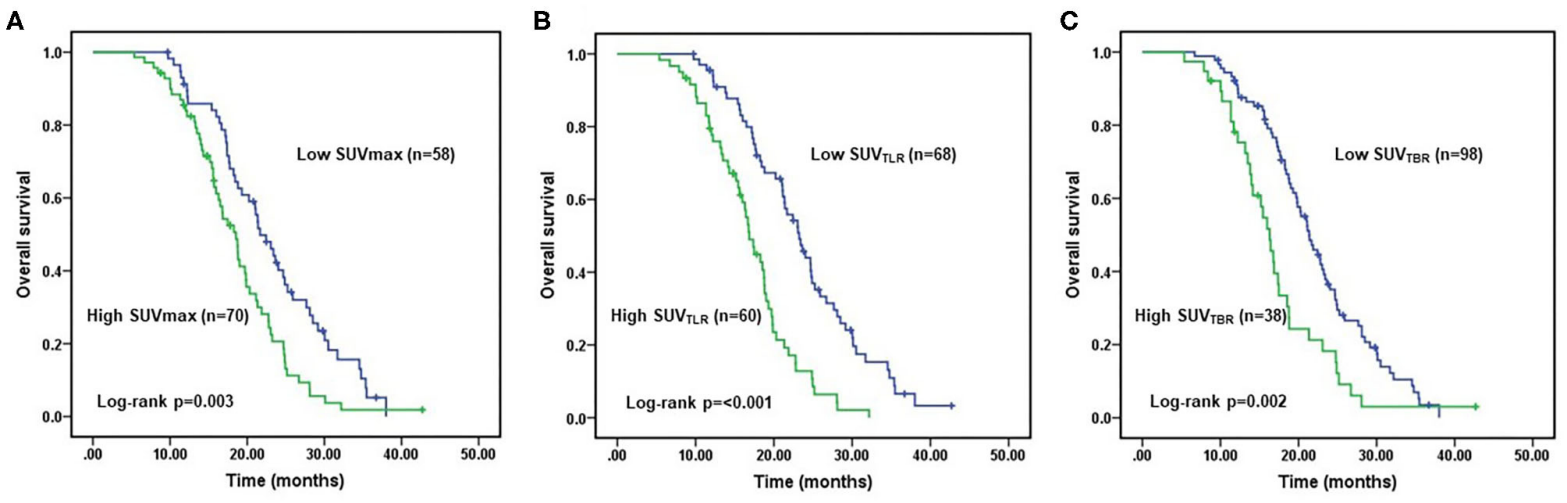
Kaplan-Meier analyses of OS according to SUVman, SUVTLR and SUV_TBR_.Patients with low SUVmax **(A)**, low SUV_TLR_ **(B)**, and low SUV_TBR_ **(C)** achieved better OS than patients with high SUVmax, high SUV_TLR_ and high SUV_TBR_.

The median follow-up time was 28 months. At the end of follow-up, 106 patients (82.8%) died from ESCC, whereas 22 patients (17.2%) were still alive. The median OS was 18.35 months (range: 5.37–42.7 months). In the univariate analysis, tumor stage (*p* = 0.020), tumor response (*p* < 0.001), SUVmax (*p* = 0.003), SUV_TLR_ (*p* < 0.001) and SUV_TBR_ (*p* = 0.002) were significantly associated with OS. As shown in Figure 2, the Kaplan-Meier curves revealed that, compared to patients in the high SUVmax group, in the high SUV_TLR_ group and in the high SUV_TBR_ group, patients in the low SUVmax group, in the low SUV_TLR_ group and in the low SUV_TBR_ group had a longer OS. None of the other parameters (i.e., age, sex, smoking history, drinking history, tumor location, T stage, and N stage) were associated with OS (Table 5). All these factors were included in the subsequent multivariate analysis. In addition, although variables such as age and gender are not significant in univariate analysis, these variables may be an important confounding factor, so they are also included in the multivariate analysis. The multivariate analysis confirmed that only tumor stage (HR = 1.777; 95% CI: 1.087–2.904, *p* = 0.022), tumor response (HR = 2.043; 95% CI: 1.170–3.567, *p* = 0.012), and SUV_TLR_ (HR = 2.620; 95% CI: 1.405–4.887, *p* = 0.002) were independent prognostic factors for OS (Figure 4). Overall, a good clinical tumor response was a favorable independent predictive factor for OS, while an advanced tumor stage and a high SUV_TLR_ were prognostic factors for poor OS. The median OS of patients in tumor stages II and III, in the OR group and non-OR group, and in the low SUV_TLR_ and high SUV_TLR_ groups were 20.68 vs. 17.77 months, 13.47 vs. 19.30 months, and 22.05 vs. 15.65 months, respectively.

**Table 5:** Univariate analysis for OS.

**Variable**	**Categories**	**HR**	**95% CI**	***p***
Age	≤ 60 vs. >60	1.122	0.751–1.677	0.573
Sex	Male vs. Female	0.858	0.536–1.375	0.525
Smoking history	Yes vs. No	0.841	0.572–1.237	0.378
Drinking history	Yes vs. No	0.793	0.541–1.162	0.234
Tumor location				
	Cervical vs. other site	0.750	0.275–2.045	0.574
	Upper thoracic vs. other site	1.021	0.632–1.651	0.932
	Mid-thoracic vs. other site	1.102	0.751–1.618	0.618
	Lower thoracic vs. other site	0.928	0.610–1.414	0.729
T stage	T1-3 vs. T4	1.014	0.637–1.616	0.952
N stage	T0 vs. T1-3	1.272	0.755–2.141	0.366
Tumor stage	IIvs. III	1.739	1.090–2.774	0.020
Tumor response	OR vs. Non-OR	0.300	0.183–0.490	<0.001
SUVmax	≤ 14.68 vs.>14.68	1.813	1.226–2.681	0.003
SUV_TLR_	≤ 4.21 vs. >4.21	2.715	1.805–4.085	<0.001
SUV_TBR_	≤ 5.06 vs. vs. >5.06	1.956	1.287–2.972	0.002

*SUV_TLR_, tumor-to-liver SUVmax ratio; SUV_TBR_, tumor-to-blood pool SUVmax ratio*.

**Figure 3:**
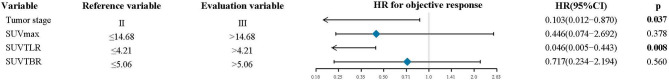
Multivarite analysis for treatment response.

**Figure 4:**
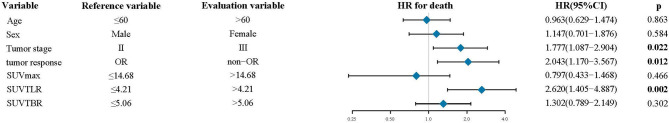
Multivarite analysis for OS.

## Discussion

Concurrent chemoradiotherapy is the main treatment for locally advanced inoperable esophageal cancer patients. In the present study, we investigated the prognostic value of SUV_TLR_ and SUV_TBR_ in locally advanced esophageal cancer patients who were treated with CCRT. We demonstrated that SUV_TLR_ was an independent predictive factor for treatment response. Furthermore, SUV_TLR_ was also an independent risk factor for OS. The median OS was significantly different below and above the cut-off value of SUV_TLR_ (22.05 vs. 15.65 months). However, SUVmax and SUV_TBR_ did not significantly correlate with OR or OS. To the best of our knowledge, this is the first study focusing on the potential prognostic role of the ratio between tumor lesions and reference background SUVmax in locally advanced esophageal cancer patients treated with CCRT.

^18^F-FDG PET/CT, which reflects glucose metabolism, has been widely applied in the management of oncological patients (10–14). The semiquantitative metabolic parameters derived from PET/CT, such as SUVmax, have been reported as useful prognostic factors in various cancers, including EC (13–15). For example, a previous retrospective study in 179 esophageal or gastroesophageal carcinoma patients showed that patients who reached a clinical complete response after definitive CCRT had a median SUVmax of 10.2, whereas those who did not achieve a clinical complete response had a median SUVmax of 15.3 (16). This study also revealed that patients with higher SUVmax were associated with poorer OS compared with patients with a lower SUVmax after definitive chemoradiotherapy. These results are supported by the study of Atsumi et al. in which the analysis of 56 esophageal carcinoma patients showed that the median SUVmax of the patients who reached a complete response was 10.6, compared with 17.6 in non-CR patients. SUVmax was significantly associated with OS, and the 2-year OS rates in the low-SUVmax (<10) and high-SUVmax (≥10) groups were 100 and 41% (15). Our results highly agree with this finding; patients with higher SUVmax (>14.68) are more likely to have a poor tumor response and a shorter OS than those with a lower one ( ≤ 14.68). Although SUVmax performs well in predicting prognosis, it has many well-known limitations, as explained in the introduction section (17–20). SUV measurements are influenced by body composition and habits, as well as time intervals. In addition, SUVmax is also affected by some technical factors, for example, errors in scanner calibration, reconstruction settings, and the attenuation-corrected protocol (33). Moreover, it was reported that FDG uptake and biodistribution are affected by tumor burden, tumor volume, and the volume of interest (34–36). In light of this variation, recent reports have suggested that applying a standardized SUVmax, i.e., tumor SUVmax normalized by an appropriate reference background, may overcome this problem in some instances (36, 37). The liver and blood pool are the most widely used reference background (37, 38), since they maintain a nearly constant SUV level over time after injecting ^18^F-FDG. There are some important advantages to applying this ratio, e.g., it is independent of management activities and weight as well as different PET/CT scanners, so at least part of the abovementioned inherent variability in SUV methodology and inter instrumental variability problems can be reduced or addressed (35, 36). Another advantage is that it is practical and simple in daily clinical use, and clinicians can easily identify primary tumor uptake compared to liver/blood pool uptake without the use of additional or special software or equipment. This method of analysis can yield reproducible results across different institutions or researchers without the need for additional imaging data that would lead to further economic burden and radiation exposure. In the present study, SUVmax of the eighth hepatic segment and aortic arch were used as the reference background to standardize one of the primary lesions, namely, SUV_TLR_ and SUV_TBR_. This methodology was reported in previous studies in lymphoma (39). In the present study, we first performed a ROC analysis to determine the best cut-off values of SUV_TLR_, SUV_TBR_ and SUVmax and then compared their predictive performance. We found that SUV_TLR_ performed the best with a significantly higher ROC of >0.85 and yielded a sensitivity and specificity of 0.957 and 0.638, respectively. SUV_TLR_ was the best predictor of the OR. Further analysis indicated that SUV_TLR_ is an independent predictor of treatment response in patients undergoing CCRT. Patients with a high SUV_TLR_ are more likely to have a poor treatment outcome than are those with a low SUVTLR. Considering OS, we demonstrated that SUV_TLR_ was also an independent predictor. With a median overall survival of 18.35 months in all patients, patients with a lower SUV_TLR_ (<4.21) had a significantly longer OS than those with a higher SUV_TLR_ (>4.21) (22.05 vs. 15.65 months). This is the first study to report the prognostic value of SUV_TLR_ in patients with esophageal cancer after CCRT. The results of this study are basically consistent with the results of previous studies in other tumors. In a study by Huang et al. (35), 504 patients with stage IIA colorectal cancer following curative resection were examined. They found that SUV_TLR_ was superior to tumor SUVmax in predicting recurrence. Patients with SUV_TLR_>6.2 revealed a 14.7-fold increased risk of disease-specific mortality. The 1-, 3-, and 5-year OS rates were 100.0, 100.0, and 98.3% for patients with lower SUV_TLR_ vs. 98.1, 83.3, and 74.3% for those with higher SUV_TLR_. Similarly, Park et al. retrospectively reviewed 167 patients with surgical margin-negative stage IA non-small cell lung cancer; the result indicated that SUV_TLR_ was an independent prognostic factor for recurrence and disease-free survival (40). In the study of 23 adenoid cystic carcinomas of salivary gland patients, high SUV_TLR_ was significantly associated with decreased OS. With a SUV_TLR_ cut-off of 2.69, there was a 1.83-fold increase in the risk of death (41). In addition, the prognostic value of SUV_TLR_ was also reported in lymphoma (30, 42). In the current study, tumor stage and treatment response were also found to be independent prognostic factors for OS, and these factors have already been well established as significant prognostic factors.

In addition, several previous studies have reported the prognostic value of the SUVmax ratio between tumor lesions and the blood pool. In a study in esophageal cancer, SUV_TBR_ was an independent prognostic factor for overall survival and distant metastasis (26). Normalization to the blood pool SUVmax improved the prognostic value and led to a higher hazard ratio than did the metabolically active tumor volume, which was also an independent prognostic factor for overall survival and distant metastasis. Similarly, Gencturk et al. (41) came to a similar conclusion that patients with lower SUV_TBR_ (<4.14) have a significantly longer progression-free survival and overall survival than those with a higher SUV_TBR_. However, in our study, Cox's proportional hazards models indicated that SUV_TBR_ was not an independent prognostic factor for OS, despite a significantly longer OS in patients with lower SUV_TBR_ in the univariate analysis. This result is highly in agreement with the previous findings by Albano et al. (43, 44). One possible explanation is that, due to the anatomical structure, the SUVmax of the blood pool may be influenced by the primary esophageal tumor and mediastinal metastatic lymph nodes. Another possible explanation is that although the SUV values of the liver and blood pool are stable over time, they are not the same in nature. The blood pool is only the storage vessel of the ^18^F-FDG, while the liver is both a storage organ and a consumption organ. This may result in different baseline states. It seems preferable to select liver uptake as a background reference. This conclusion was supported by Itti E's previous result that with mediastinal blood pool as a reference, PET was unable to distinguish early responders from nonresponders (45). In contrast, with liver as a reference, 2-year progression-free survival was significantly different between patients with PET-negative findings and patients with PET-positive findings. Therefore, we suggest that the liver is a more optimal reference background tissue than the blood pool for normalization of FDG uptake when performing semiquantitative metabolic tumor response and prognosis prediction.

This study has some limitations. First, this is a retrospective single-center study. Further prospective, multicenter, clinical trial should be conducted to clarify the results of our findings. Second, although we have applied endoscopic ultrasonography, enhanced CT and PET/CT, clinical TNM staging is still not as accurate as pathological TNM staging. Lastly, only a small number of patients underwent a second PET/CT examination after the end of treatment, so we did not have enough data to deeply analyze the changes in metabolic parameters before and after treatment, as well as the prognostic value.

## Conclusion

In summary, our study indicated that the PET-derived tumor-to-liver maximum standardized uptake value ratio (SUV_TLR_) is superior to the tumor SUVmax and tumor-to-blood pool maximum standardized uptake value ratio (SUV_TBR_) in predicting treatment and overall survival in patients with ESCC undergoing first-line chemoradiotherapy. Patients with a higher SUV_TLR_ are more likely to have a poor treatment response and shorter overall survival than those with a lower one.

## Data Availability Statement

The datasets generated for this study are available on request to the corresponding author.

## Ethics Statement

The studies involving human participants were reviewed and approved by The ethics committee of Shandong Cancer Hospital and Institute approved the study. Written informed consent for participation was not required for this study in accordance with the national legislation and the institutional requirements.

## Author Contributions

ML and YS: conceptualization, methodology, supervision, and project administration. YH and LM: software. CW and KZ: formal analysis, writing-original draft preparation, and writing-review and editing. SH: resources. KZ: data curation. All authors contributed to the article and approved the submitted version.

## Conflict of Interest

The authors declare that the research was conducted in the absence of any commercial or financial relationships that could be construed as a potential conflict of interest.

## References

[1] Siegel RL Miller KD Jemal A Cancer statistics. Cancer J Clin. (2019) 69:7–34. 10.3322/caac.21551 30620402

[2] Enzinger PC Mayer RJ. Esophageal cancer. N Engl J Med. (2003) 349:2241–52. 10.1056/NEJMra035010 14657432

[3] Courrech Staal EF van Coevorden F Cats A Aleman BM van Velthuysen ML Boot H. Outcome of low-volume surgery for esophageal cancer in a high-volume referral center. Ann Surg Oncol. (2009) 16:3219–26. 10.1245/s10434-009-0700-5 19777184

[4] Cooper JS Guo MD Herskovic A Macdonald JS Martenson JA Jr Al-Sarraf M. Chemoradiotherapy of locally advanced esophageal cancer: long-term follow-up of a prospective randomized trial (RTOG 85-01). Radiation Therapy Oncology Group. JAMA. (1999) 281:1623–7. 10.1001/jama.281.17.1623 10235156

[5] Zhao T Chen H Zhang T. Docetaxel and cisplatin concurrent with radiotherapy versus 5-fluorouracil and cisplatin concurrent with radiotherapy in treatment for locally advanced oesophageal squamous cell carcinoma: a randomized clinical study. Med Oncol. (2012) 29:3017–23. 10.1007/s12032-012-0228-6 22476809

[6] Conroy T Galais M-P Raoul J-L Bouché O Gourgou-Bourgade S Douillard J-Y. Definitive chemoradiotherapy with FOLFOX versus fluorouracil and cisplatin in patients with oesophageal cancer (PRODIGE5/ACCORD17): final results of a randomised, phase 2/3 trial. Lancet Oncol. (2014) 15:305–14. 10.1016/s1470-2045(14)70028-2 24556041

[7] Zhu Y Zhang W Li Q Li Q Qiu B Liu H. A phase II randomized controlled trial: definitive concurrent chemoradiotherapy with docetaxel plus cisplatin versus 5-fluorouracil plus cisplatin in patients with oesophageal squamous cell carcinoma. J Cancer. (2017) 8:3657–66. 10.7150/jca.20053 29151952 PMC5688918

[8] Minsky BD Neuberg D Kelsen DP Pisansky TM Ginsberg R Benson A. Neoadjuvant chemotherapy plus concurrent chemotherapy and high-dose radiation for squamous cell carcinoma of the esophagus: a preliminary analysis of the phase II intergroup trial 0122. J Clin Oncol. (1996) 14:149–55. 10.1200/JCO.1996.14.1.149 8558190

[9] Kato H Kuwano H Nakajima M Miyazaki T Yoshikawa M Ojima H. Comparison between positron emission tomography and computed tomography in the use of the assessment of esophageal carcinoma. Cancer. (2002) 94:921–8. 10.1002/cncr.10330 11920459

[10] Gallamini A Zwarthoed C Borra A. Positron emission tomography (PET) in oncology. Cancers. (2014) 6:1821–89. 10.3390/cancers6041821 25268160 PMC4276948

[11] Noble F Bailey D Panel SUGT Tung K Byrne JP. Impact of integrated PET/CT in the staging of oesophageal cancer: a UK population-based cohort study. Clin Radiol. (2009) 64:699–705. 10.1016/j.crad.2009.03.003 19520214

[12] Bar-Shalom R Guralnik L Tsalic M Leiderman M Frenkel A Gaitini D. The additional value of PET/CT over PET in FDG imaging of oesophageal cancer. Eur J Nucl Med Mol Imag. (2005) 32:918–24. 10.1007/s00259-005-1795-y 15838691

[13] Chang S Kim SJ. Prediction of recurrence and mortality of locally advanced esophageal cancer patients using pretreatment F-18 FDG PET/CT parameters: intratumoral heterogeneity, SUV, and volumetric parameters. Cancer Biother Radiopharm. (2016) 31:1–6. 10.1089/cbr.2015.1932 26844846

[14] Barber TW Duong CP Leong T Bressel M Drummond EG Hicks RJ. 18F-FDG PET/CT has a high impact on patient management and provides powerful prognostic stratification in the primary staging of esophageal cancer: a prospective study with mature survival data. J Nucl Med. (2012). 53:864–71. 10.2967/jnumed.111.101568 22582047

[15] Atsumi K Nakamura K Abe K Hirakawa M Shioyama Y Sasaki T. Prediction of outcome with FDG-PET in definitive chemoradiotherapy for esophageal cancer. J Radia Res. (2013) 54:890–8. 10.1093/jrr/rrt021 23520267 PMC3766293

[16] Suzuki A Xiao L Hayashi Y Macapinlac HA Welsh J Lin SH Prognostic significance of baseline positron emission tomography and importance of clinical complete response in patients with esophageal or gastroesophageal junction cancer treated with definitive chemoradiotherapy. Cancer. (2011) 117:4823–33. 10.1002/cncr.26122 21456015 PMC3144261

[17] Adams MC Turkington TG Wilson JM Wong TZ. A systematic review of the factors affecting accuracy of SUV measurements. Am J Roentgenol. (2010) 195:310–20. 10.2214/AJR.10.4923 20651185

[18] Boellaard R Krak NC Hoekstra OS Lammertsma AA. Effects of noise, image resolution, and ROI definition on the accuracy of standard uptake values: a simulation study. J Nucl Med. (2004) 45:1519–27. 15347719

[19] Boktor RR Walker G Stacey R Gledhill S Pitman AG. Reference range for intrapatient variability in blood-pool and liver SUV for 18F-FDG PET. J Nucl Med. (2013) 54:677–82. 10.2967/jnumed.112.108530 23512357

[20] Weber WA Gatsonis CA Mozley PD Hanna LG Shields AF Aberle DR. Repeatability of 18F-FDG PET/CT in advanced non-small cell lung cancer: prospective assessment in 2 multicenter trials. J Nucl Med. (2015) 56:1137–43. 10.2967/jnumed.114.147728 25908829 PMC4699428

[21] Sprinz C Altmayer S Zanon M Watte G Irion K Marchiori E. Effects of blood glucose level on 18F-FDG uptake for PET/CT in normal organs: A systematic review. PLoS ONE. (2018) 13:e0193140. 10.1371/journal.pone.0193140 29486008 PMC5828444

[22] Albano D Giubbini R Bertagna F. 18F-FDG PET/CT in splenic marginal zone lymphoma. Abdom Radiol. (2018). 43:2721–7. 10.1007/s00261-018-1542-z 29500652

[23] Albano D Mazzoletti A Spallino M Muzi C Zilioli VR Pagani C. Prognostic role of baseline 18F-FDG PET/CT metabolic parameters in elderly HL: a two-center experience in 123 patients. Ann Hematol. (2020) 99:1321–30. 10.1007/s00277-020-04039-w 32333153

[24] Wahl RL Jacene H Kasamon Y Lodge MA. From RECIST to PERCIST: Evolving Considerations for PET response criteria in solid tumors. J Nucl Med. (2009) 50 Suppl 1:122S−50S. 10.2967/jnumed.108.057307 19403881 PMC2755245

[25] Keramida G Dizdarevic S Bush J Peters AM Quantification of tumour (18) F-FDG uptake: Normalise to blood glucose or scale to liver uptake? Eur Radiol. (2015) 25:2701–8. 10.1007/s00330-015-3659-6 25899414

[26] Butof R Hofheinz F Zophel K Stadelmann T Schmollack J Jentsch C. Prognostic value of pretherapeutic tumor-to-blood standardized uptake ratio in patients with esophageal carcinoma. J Nucl Med. (2015) 56:1150–6. 10.2967/jnumed.115.155309 26089549

[27] Law WP Maggacis N Jeavons SJ Miles KA. Concordance of 18F-FDG PET uptake in tumor and normal tissues on PET/MRI and PET/CT. Clin Nucl Med. (2017) 42:180–6. 10.1097/RLU.0000000000001514 28033217

[28] Lee JW Hwang SH Kim DY Han KH Yun M. Prognostic value of FDG uptake of portal vein tumor thrombosis in patients with locally advanced hepatocellular carcinoma. Clin Nucl Med. (2017) 42:e35–e40. 10.1097/RLU.0000000000001422 27775940

[29] Park SY Cho A Yu WS Lee CY Lee JG Kim DJ. Prognostic value of total lesion glycolysis by 18F-FDG PET/CT in surgically resected stage IA non-small cell lung cancer. J Nucl Med. (2015) 56:45–9. 10.2967/jnumed.114.147561 25525185

[30] Annunziata S Cuccaro A Calcagni ML Hohaus S Giordano A Rufini V. Interim FDG-PET/CT in Hodgkin lymphoma: the prognostic role of the ratio between target lesion and liver SUVmax (rPET). Ann Nucl Med. (2016) 30:588–92. 10.1007/s12149-016-1092-9 27246952

[31] Hasenclever D Kurch L Mauz-Korholz C Elsner A Georgi T Wallace H. qPET - a quantitative extension of the Deauville scale to assess response in interim FDG-PET scans in lymphoma. Eur J Nucl Med Mol Imag. (2014) 41:1301–8. 10.1007/s00259-014-2715-9 24604592

[32] Ko KH Hsu HH Huang TW Gao HW Cheng CY Hsu YC. Predictive value of 18F-FDG PET and CT morphologic features for recurrence in pathological stage IA non-small cell lung cancer. Medicine. (2015) 94:e434. 10.1097/MD.0000000000000434 25621697 PMC4602644

[33] Lim CH Moon SH Cho YS Choi JY Lee KH Hyun SH. Prognostic value of (18)F-fluorodeoxyglucose positron emission tomography/computed tomography in patients with combined hepatocellular-cholangiocarcinoma. Eur J Nucl Med Mol Imag. (2019) 46:1705–12. 10.1007/s00259-019-04327-2 31049603

[34] Viglianti BL Wale DJ Wong KK Johnson TD Ky C Frey KA. Effects of tumor burden on reference tissue standardized uptake for PET imaging: modification of PERCIST criteria. Radiology. (2018) 287:993–1002. 10.1148/radiol.2018171356 29558296

[35] Huang J Huang L Zhou J Duan Y Zhang Z Wang X. Elevated tumor-to-liver uptake ratio (TLR) from (18)F-FDG-PET/CT predicts poor prognosis in stage IIA colorectal cancer following curative resection. Eur J Nucl Med Mol Imag. (2017) 44:1958–68. 10.1007/s00259-017-3779-0 28812134 PMC5656694

[36] Hofheinz F Butof R Apostolova I Zophel K Steffen IG Amthauer H. An investigation of the relation between tumor-to-liver ratio (TLR) and tumor-to-blood standard uptake ratio (SUR) in oncological FDG PET. EJNMMI Res. (2016) 6:19. 10.1186/s13550-016-0174-y 26936768 PMC4775714

[37] Chiaravalloti A Danieli R Abbatiello P Di Pietro B Travascio L Cantonetti M. Factors affecting intrapatient liver and mediastinal blood pool (1)(8)F-FDG standardized uptake value changes during ABVD chemotherapy in Hodgkin's lymphoma. Eur J Nucl Med Mol Imag. (2014) 41:1123–32. 10.1007/s00259-014-2703-0 24562647

[38] Park J Chang KJ Seo YS Byun BH Choi JH Moon H. Tumor SUVmax normalized to liver uptake on (18)F-FDG PET/CT predicts the pathologic complete response after neoadjuvant chemoradiotherapy in locally advanced rectal cancer. Nucl Med Mol Imag. (2014) 48:295–302. 10.1007/s13139-014-0289-x 26396634 PMC4571668

[39] Albano D Bertoli M Ferro P Fallanca F Gianolli L Picchio M. 18F-FDG PET/CT in gastric MALT lymphoma: a bicentric experience. Eur J Nucl Med Mol Imag. (2017) 44:589–97. 10.1007/s00259-016-3518-y 27619357

[40] Park HL Yoo IR Boo SH Park SY Park JK Sung SW. Does FDG PET/CT have a role in determining adjuvant chemotherapy in surgical margin-negative stage IA non-small cell lung cancer patients? J Cancer Res Clin Oncol. (2019) 145:1021–6. 10.1007/s00432-019-02858-7 30756189 PMC11810241

[41] Gencturk M Ozturk K Koksel Y Li F Cayci Z. Pretreatment quantitative (18)F-FDG PET/CT parameters as a predictor of survival in adenoid cystic carcinoma of the salivary glands. Clin Imag. (2019) 53:17–24. 10.1016/j.clinimag.2018.09.021 30308429

[42] Annunziata S Cuccaro A Tisi MC Hohaus S Rufini V. FDG-PET/CT at the end of immuno-chemotherapy in follicular lymphoma: the prognostic role of the ratio between target lesion and liver SUVmax (rPET). Ann Nucl Med. (2018) 32:372–7. 10.1007/s12149-018-1243-2 29464479

[43] Albano D Bosio G Pagani C Re A Tucci A Giubbini R. Prognostic role of baseline 18F-FDG PET/CT metabolic parameters in Burkitt lymphoma. Eur J Nucl Med Mol Imag. (2019) 46:87–96. 10.1007/s00259-018-4173-2 30276438

[44] Albano D Bosio G Bianchetti N Pagani C Re A Tucci A. Prognostic role of baseline 18F-FDG PET/CT metabolic parameters in mantle cell lymphoma. Ann Nucl Med. (2019) 33:449–58. 10.1007/s12149-019-01354-9 30929200

[45] Itti E Juweid ME Haioun C Yeddes I Hamza-Maaloul F El Bez I. Improvement of early 18F-FDG PET interpretation in diffuse large B-cell lymphoma: importance of the reference background. J Nuc Med. (2010) 51:1857–62. 10.2967/jnumed.110.080556 21078789

